# From Immune Dysregulations to Therapeutic Perspectives in Myelodysplastic Syndromes: A Review

**DOI:** 10.3390/diagnostics11111982

**Published:** 2021-10-26

**Authors:** Thibault Comont, Emmanuel Treiner, François Vergez

**Affiliations:** 1 Department of Internal Medicine, IUCT-Oncopole, Toulouse University Hospital (CHU-Toulouse), 31300 Toulouse, France; 2 Cancer Research Center of Toulouse, Unité Mixte de Recherche (UMR) 1037 INSERM, ERL5294 Centre National de La Recherche Scientifique, 31100 Toulouse, France; vergez.Francois@iuct-oncopole.fr; 3 School of Medicine, Université Toulouse III—Paul Sabatier, 31062 Toulouse, France; treiner.e@chu-toulouse.fr; 4 Laboratory of Immunology, Toulouse University Hospital (CHU-Toulouse), 31300 Toulouse, France; 5 Infinity, Inserm UMR1291, 31000 Toulouse, France; 6 Laboratory of Hematology, IUCT-Oncopole, Toulouse University Hospital (CHU-Toulouse), 31300 Toulouse, France

**Keywords:** myelodysplastic syndromes, immune, inflammation, T-cell

## Abstract

The pathophysiology of myelodysplastic syndromes (MDSs) is complex and often includes immune dysregulation of both the innate and adaptive immune systems. Whereas clonal selection mainly involves smoldering inflammation, a cellular immunity dysfunction leads to increased apoptosis and blast proliferation. Addressing immune dysregulations in MDS is a recent concept that has allowed the identification of new therapeutic targets. Several approaches targeting the different actors of the immune system have therefore been developed. However, the results are very heterogeneous, indicating the need to improve our understanding of the disease and interactions between chronic inflammation, adaptive dysfunction, and somatic mutations. This review highlights current knowledge of the role of immune dysregulation in MDS pathophysiology and the field of new drugs.

## 1. Introduction

Myelodysplastic syndromes (MDSs) are acquired clonal myeloid malignancies characterized by ineffective hematopoiesis resulting in peripheral cytopenia and a risk of progression to acute myeloid leukemia (AML) [1]. MDSs are a heterogeneous group of diseases with differences in clinical presentation, biological features, prognosis, and treatment. Severity is defined by the Revised International Prognostic Scoring System (IPSS-R) [2] but some studies identified other prognostic factors such as patient characteristics (comorbidities) [3] or somatic mutations [4] and new scores are actually being developed [5].

In Low-risk MDS (LR-MDS), the objectives of treatments are to correct peripheral cytopenia. In contrast, blastic progression and risk of AML transformation are the major concerns of High-Risk MDS (HR-MDS) patients, and treatments target the malignant clone [6]. In both situations, however, available therapeutic options are limited, and a better comprehension of MDS pathophysiology is needed to identify new targets [7].

The central role of the immune system in tumor surveillance is well known and targeted in solid tumors [8] but its implication in myeloid malignancies pathogenesis, especially in MDS, is less well described. However, recent studies tried to explore dysregulations in both innate and adaptive compartments of immunity and their consequences on MDS presentation, prognosis, and risk of AML transformation. Moreover, T-cells from patients with MDS may originate from the malignant clone [9]. Together, these studies showed that MDS is associated with several immune alterations leading to cytopenia and/or disease progression; further, this immune dysregulation is a dynamic process, evolving during the disease [10].

Thus, an understanding of these immunological disorders may allow for improved initial classification of patients but also for innovative and more targeted treatments.

In this review, we will describe the current knowledge on the immune dysregulations involved in the early or late steps of MDS pathophysiology, their clinical implications, prognosis, and their present and potential therapeutic actions.

## 2. Inflamm-Aging as a Risk for Clonal Hematopoiesis

MDS diagnoses increase with advanced age [11]. Indeed, whereas prevalence is about 4/100,000 in the US population, it increases after 60 years to 35/100,000, suggesting the role of aging in disease initiation. Aging is now a well-understood process with several alterations in different systems including immune and hematopoietic compartments. Consequences include increased risk of infections, auto-immunity, and/or malignancies. Several molecular mechanisms are involved in aging, including genomic damage, telomere curtailment, or epigenetic alterations [12]. Furthermore, aging is associated with an increase in the number of somatic mutations, which, in the hematopoietic compartment, create a broad array of genetically distinct stem cells (named clones).

Clonal hematopoiesis is characterized by the over-representation of blood cells derived from a single clone. Several studies have identified recurrent somatic mutations in several genes driving clonal hematopoiesis. These loss-of-function mutations mostly concern genes involved in DNA methylation (*DNA methyl-transferase 3A*, *DNMT3A* and *Ten-eleven-translocation 2*, *TET2*), chromatin regulation (*ASXL1*), or splicing factors (*Splicing Factor 3b Subunit 1*, *SF3B1*, *Serine and Arginine Rich Splicing Factor 2*, *SRSF2*, and *U2 Small Nuclear RNA Auxiliary Factor 1*, *U2AF1*) [13,14,15,16,17,18,19,20,21]. The term “clonal hematopoiesis of indeterminate potential” (CHIP) was then introduced to distinguish non-malignant clonal hematopoiesis, which is clearly linked to cancer-associated mutations from other forms of clonal hematopoiesis. CHIP is defined in a subset of individuals with clonal hematopoiesis based on a variant allele frequency ≥ 2% of a somatic mutation in a hematologic malignancy-associated gene. Individuals with CHIP have a modestly increased long-term risk of developing hematologic malignancies. Somatic clones increase in frequency with each decade of life (from 1% of healthy individuals under the age of 40 to 10–20% after 70 years old). CHIP affects 20–40% of individuals > 80 years, and is associated with an increased risk for transformation to MDS or AML [20,22,23,24,25].

Whereas the molecular defects driving CHIP are well studied, how these alterations occur is less well understood. Environmental stimuli and stressors, including chronic infections and inflammatory processes, may play a key role in CHIP [26]. Indeed, aging is also characterized by immune senescence with quantitative and qualitative modifications in several cell types. A major characteristic of immune-aging is chronic inflammation (by persistent infection or a sterile inflammatory process) [27]. This phenotype notably includes elevated levels of pro-inflammatory cytokines (TNF-α, interleukin(IL)-1β, IL-6, RANTES). Some studies explored the consequences of chronic inflammation in hematopoietic stem cells (HSCs) in the context of aging and showed a depletion of HSCs [28,29] or functional alterations [30,31]. Moreover, chronic inflammation favors the expansion of HSCs with CHIP mutations [32]. Inflammation can initiate the CHIP process but also its progression. Indeed, in vivo studies showed that CHIP-associated mutations, such as *TET2* or *DNMT3A*, could confer a pro-inflammatory profile, with high levels of TNF-α, IL-1, IL-6, IL-8, or IFN-γ that could drive the expansion of clonal HSCs [32,33].

Taken together, chronic inflammation could participate in CHIP initiation, and mutant clones promote this pro-inflammatory profile, driving clonal expansion.

## 3. Smoldering Inflammation Acts Early in MDS Pathophysiology

We previously described the role of inflammation in clonal selection and progression, but dysregulations of innate immune system components are also involved in MDS pathophysiology.

### 3.1. Proinflammatory Cytokines

Several studies observed high levels of proinflammatory cytokines in MDS patients [34], including TNF-α [35,36], IFN-γ [37], TGF-β, IL-6 [38], or IL-8 [39] with a potential independent risk factor of progression [40]. Moreover the levels of TNF-α, IFN-γ, and IL-6 are associated with apoptosis induction in bone marrow (BM) [41,42]. This can explain why this cytokine profile is more frequently observed in LR-MDS, while immunosuppressive cytokines such as IL-10 are more prevalent in HR-MDS [43].

### 3.2. NLRP3 Inflammasome

Inflammasomes play a key role in MDS pathophysiology, particularly in LR-MDS [44,45,46]. In an activated NLR family, NOD-like receptor pyrin domain-containing 3 (NLRP3) complexes are implicated in the pyroptosis of MDS cells, which is an inflammatory cell death process [47]. The NLRP3–pyroptosis axis is found to be activated in MDS BM cells [48] and is responsible for LR-MDs features such as macrocytosis and ineffective hematopoiesis resulting in cytopenia. Activation of the NLRP3 inflammasome is initiated by damage-associated molecular pattern signals (DAMP) (e.g., S100A8/9 [49]), and involves signal transducers such as serine/threonine kinases IRAK1/4 [50] and the E3 ubiquitin ligase TRAF6. Once activated, NLRP3 recruits and activates pro-caspase-1, which then activates pro-IL-1β, pro-IL-18, and the pore-forming protein gasdermin D [51], followed by pyroptotic cell death with the release of active IL-1β, IL-18, and other intracellular proteins that contribute to local inflammation [48,52]. Interestingly, S100A8 and S100A9 proteins are increased in the HSCs or blood of MDS patients, especially in LR-MDS [53]. These proteins also expand Myeloid-Derived Suppressor Cells (MDSCs) in the BM of patients with MDS [54].

### 3.3. Toll-like-Receptors (TLR)

The TLR family contains 10 subtypes in humans, and these are expressed on a variety of hematopoietic cell types [55]. TLR signaling regulates hematopoietic stem and progenitor cell function [56]. MDS HSCs are particularly sensitive to pyroptosis because of high levels of TLR (TLR 1-2-6) expression and activation [42,57,58,59]. Moreover, while the overexpression of TLR-2 or TLR-4 is observed in LR-MDS, increased expression of TLR-6 correlates with HR disease [60]. HSCs of LR-MDS patients are more sensitive to TLR ligands (DAMP) and some of these ligands are more abundant in the BM and/or serum of MDS patients compared to healthy controls (ex HMGB1 [61], S100A8/9 [53]). In LR-MDS, S100A8/A9 is more abundant (also secreted by MDSCs) and binds to TLR-4 and CD33, stimulating inflammasome assembly and pyroptotic cell death by promoting the production of inflammasome components, Reactive Oxygen Species (ROS), and proinflammatory cytokines such as IL-1β and IL-18, and the expansion of MDSCs. The consequence is the suppression of normal hematopoiesis via the production of cytokines such as IL-10 and TGF-β.

### 3.4. Immune Cells

MDSCs [62] are increased in the BM and blood of MDS patients compared to healthy donors, especially in HR-MDS, and are involved in ineffective hematopoiesis. MDSCs also induce T-cell immunosuppression via the secretion of IL-10 and TGF-β, leading to MDS progression. MDSC expansion is driven by the interaction of S100A9 with CD33.

Monocytes: The number of monocytes in the blood or BM of MDS patients is increased, especially in HR-MDS, but their ability to differentiate into macrophages to then use their phagocytic function is decreased [63,64]. Cytokines, chemokines, and TLR genes are also downregulated in BM monocytes from MDS patients. Moreover, as the disease progresses, the number of abnormal monocytes increases in the BM of the patients [65]. In addition, the ratio of M2 macrophages to monocytes is higher in patients with MDS whereas the ratio of M1 to M2 macrophages is lower in MDS patients. Because monocytes play a key role in the defense against microbial agents, these alterations may explain the susceptibility to infections observed in MDS patients.

Dendritic cells (DCs): Some studies described quantitative or qualitative defects of DCs in MDS patients [66] with, for example, an altered cytokine profile [67]. High secretion of IL-10 may contribute to the immunosuppressive phenotype. Interestingly, it was shown that cytogenetic abnormalities in DCs from MDS patients were similar to the malignant clones of MDS, suggesting that they originated from the malignant clones [68]. DCs derived from MDS monocytes also have morphologic and phenotypic abnormalities [64].

Lymphocytes: A reduced number of lymphocytes, or their dysfunction, can also have prognostic implications. For example, the number of Tregs could be a prognostic factor in LR-MDS and predict the severity of anemia, AML transformation, and overall survival [69]. A low number of NK-cells, their reduced cytotoxic capacity, or their Killer-cell immunoglobulin-like receptors (KIR) gene haplotype are also associated with poor prognosis and AML transformation [70,71,72].

## 4. Positive and Negative Immune Regulators

Immune cell signaling includes co-stimulatory and co-inhibitory (named Immune check points or ICPs) molecules that are differentially expressed by a variety of cell types (such as antigen-presenting cells, B-cells, macrophages, and T-cells) and may be regulated by activation [73]. Some of these molecules were studied in MDS and appeared to be potential therapeutic targets.

### 4.1. Co-Stimulatory Molecules

- OX40, Inducible T-cell COStimulator (ICOS) and 4-1BB+: *Tumor protein 3 (TP53)* MDS patients display reduced numbers of OX40+ cytotoxic T-cells and helper T-cells, as well as reduced ICOS+ and 4-1BB+ NK-cells [74].
- Other positive regulators, such as CD244, CD80, and CD40, have been studied but their implication is not clearly validated and they are not yet interesting therapeutic leads [75,76].

### 4.2. Coinhibitory Molecules

Cytotoxic T-Lymphocyte Antigen 4 (CTLA-4): Upregulation of CTLA-4 has been observed in CD34+ cells from HR-MDS, and levels further increased in the post hypomethylating agents (HMA) failure setting [77].

Program cell death-1, and L1 (PD-1 and PD-L1): The PD-1/PD-L1 axis is well studied. Upregulation of PD-1 and PDL-1 was seen in CD34+ BM cells from patients with MDS and in T-cells, with further upregulation seen in those previously treated with HMAs [77,78]. Moreover, a higher rate of PD-L1 expression on BM blasts and HSCs from *TP53*-mutated AML and MDS patients is observed [74]. The expression of ICPs could be upregulated in the presence of pro-inflammatory cytokines [79] or in response to S100A9 signaling, contributing to MDSC-induced HSC death [80].

T-cell immunoglobulin and mucin-containing protein-3 (TIM3): Some studies showed that TIM3 is overexpressed in HSCs, blasts, and CD8-T-cells from MDS patients [81,82]. Excessive TIM3+ HSCs are closely related to disease severity. TIM3+ HSCs and T-cells displayed aberrant functions [83]. Moreover, MDSCs suppress CD8+ T cells through the TIM3/Galectin 9 pathway [84].

T cell immunoreceptor with Ig and ITIM domains (TIGIT): TIGIT was found to be highly expressed in NK-cells and T-cells from the blood of MDS patients with an association with disease progression [85]. The overexpression of TIGIT can be associated with decreased NK and T-cell functions, and lower secretions of activating cytokines such as IFN-γ and TNF-α.

ICPs expression is also associated with resistance to hypomethylating agents [77].

Moreover, the impact of ICP expression on MDS prognosis was assessed in a recent study, which evaluated the mutation burden in genes coding for ICP molecules (LAG-3, CTLA-4, B7H3, PD-1, PD-L1, etc.). The authors observed a high prevalence of mutations in these genes, with an impact on overall survival [86].

## 5. Opposite Adaptive Dysfunctions in LR and HR-MDS

In the past few years, several studies have explored the role of adaptive immunity in MDS pathogenesis and have highlighted differences in immune profiles between LR and HR-MDS. While LR-MDS presents with a more inflammatory and cytotoxic profile, HR-MDS is characterized by a more suppressive microenvironment [10]. For example, whereas IL-17-producing CD4+ T-cells from LR-MDS patients are increased and Tregs number and function are reduced [87], HR-MDS patients display quantitative and qualitative alterations of CD8+ T-cells [88], NK-cells [70], and an expansion of Tregs [89,90] and MDSCs. Moreover, HR-MDS patients overexpress ICPs on their blasts (such as PD-L1 or TIM-3) [83,91,92], their HSCs (PD-L1, TIM-3, or CD47) [77,78,81,93], their T and NK-cells (PD-1, TIGIT, or TIM-3) [78,82,84,85], or their macrophages [94].

## 6. Immune Phenotypes Can Be Associated with Somatic Mutations

Clonal hematopoiesis and MDS progression are driven by the acquisition of somatic mutations. However, these mutations not only act on clonal selection but also on the immune microenvironment. Then, mutations affecting DNA methylation (*TET-2* and *DNMT3A*), histone modification (*ASXL1*), or splicing factors (*SF3B1*, *SRSF2*, *U2AF1*) have been associated with increased inflammation in patients with CHIP or MDS.

*TET2* mutation seems to promote clonal HSC dominance by creating an inflammatory environment [32,95]. Interestingly, *TET2* mutant CHIP is more highly associated with elevated IL-6, while *DNMT3A* mutant CHIP is more highly associated with elevated TNF-α or IFN-γ [96,97]. Recently Zhao et al. found that *TET2* or *IDH 1/2* mutations were more frequent in MDS patients presenting with systemic autoinflammatory or autoimmune diseases [98]. In this study, *TET2/IDH* mutant patients’ phenotyping showed a reduction of Tregs and deep alterations in CD8+ T-cell distribution.

Spliceosome mutations (such *SF3B1*, *U2AF1*, and *SRSF2*) have also been associated with immune dysregulation such as TRL activity [99,100] or proinflammatory cytokine production [101].

As previously described, Sallman et al. recently described a distinct immune phenotype associated with *TP53* mutated MDS/AML patients [74]. These patients overexpressed ICPs PD-L1 (especially in HSCs) and CTLA-4, associated with poor survival. Moreover, *TP53* mutant patients displayed reduced numbers of BM OX40+ cytotoxic T-cells and helper T-cells, reduced ICOS+ and 4-1BB+ NK cells, and an increased number of immunosuppressive regulatory T-cells and MDSCs. Finally, they found that a higher proportion of BM infiltrating ICOShigh/PD-1neg Tregs was a highly significant predictor of overall survival.

Somatic mutations not involved in clonal hematopoiesis can also drive inflammatory manifestations and MDS. Recently, an autoinflammatory disease characterized by a somatic mutation of *UBA1* has been described and named VEXAS (Vacuoles, E1 Enzyme, X-linked, Autoinflammatory, Somatic syndrome) [102]. Systemic manifestations often include neutrophilic skin lesions, polychondritis, pulmonary infiltrates, or thrombosis [103]. VEXAS can be associated with hematological disorders, especially MDS in 25–55% of cases [102,104,105,106,107]. Implication of *ubiquitin-like modifier activating enzyme 1* (*UBA1)* mutation in MDS pathophysiology is not well studied but could be explained by chronic inflammation.

Finally, driver mutations have been found in lymphoid precursors, raising the question of the potential impact of these mutations on immune responses in the MDS context [108].

## 7. Immune Strategies in MDS: Past, Present and Future

Targeting the immune system in MDS is not a novel concept, but modern approaches such as ICP inhibitors (ICPi) or an adoptive T-cell transfer may be promising and are still under evaluation (Table 1).

### 7.1. “Old” Treatments

Immunosuppressive and immunomodulatory therapies have been used in selected MDS patients.

First, allogenic Hematopoietic stem-cell transplantation (HSCT) is actually the only curative treatment for MDS. It is considered an immune approach because of the graft versus leukemia effect but also because of its immune modulatory properties, especially in the context of reduced-intensity conditioning transplant programs [109].

The first and most commonly used immunosuppressive treatments were antithymocyte globulin and ciclosporin. Studies have reported hematologic improvement in 20–60% of selected MDS patients (young age, hypoplastic LR-MDS, normal karyotype), especially when used in combination [110,111,112]. These responses seem to be associated with lower rates of AML transformation [111,113,114,115].

In del(5q) LR-MDS, lenalidomide, an immunomodulatory drug, has shown a high rate of efficacy with sustained responses [116]. It acts on T-cell activity via several mechanisms, including reduced T-cell tolerance and increased effector functions [117]. Other studies have shown its role in the ubiquitination and degradation of specific substrates [118]. Lenalidomide is currently used in LR-MDS with del(5) patients who are still transfusion-dependent despite treatment with erythropoietic-stimulating agents [119].

Alemtuzumab is a humanized monoclonal antibody directed against CD52, a glycosyl phosphatidyl inositol-anchored cell surface marker abundantly expressed on B and T-cells and at low levels on effector cells of the innate immune system. It was evaluated in a subset of MDS patients and showed good efficacy and a good safety profile [120].

Targeting pro-inflammatory cytokines is also an option in MDS, especially in LR-MDS patients. Infliximab, an antibody targeting TNF-α, was evaluated in LR-MDS and showed a moderate hematologic response but a good safety profile [121]. Other agents such as Etanercept, were associated with HMA and showed interesting results [122]. Studies using the anti-TNF-α antibody in combination with other drugs are ongoing. Other biologics are actually used in MDS-associated systemic inflammatory or auto-immune disorders (such as anti-IL-1, IL-6 antibodies) but are also evaluated in combination with other drugs [123].

### 7.2. Targeting TGF-β Superfamily in LR-MDS with Anemia

Increased concentrations of TGF-β superfamily ligands, including growth differentiation factor 11 (GDF11) in bone marrow have been linked to ineffective erythropoiesis in MDS [124,125]. Luspatercept (ACE-536) is a soluble fusion protein that binds to GDF11 and other TGF-β superfamily ligands. It acts as an activin receptor type II ligand trap and allows the restoration of terminal maturation of erythroid progenitors by diminishing Smad2/3 signaling. It was recently evaluated in a phase III, randomized, double blind, placebo-controlled study for LR-MDS patients with ring sideroblasts who were refractory/intolerant or ineligible for ESA, and transfusion dependent. Results are encouraging, and luspartecept obtained FDA and EMA approbation in this indication [126,127].

### 7.3. Targeting the Inflammasomes

As stated before, inflammasomes play a key role in dysregulated innate immune signaling in MDS, and targeting key hub mediators could be a promising option. New molecules such as CA-4948 (IRAK4 inhibitor), ibrutinib (BTK inhibitor acting as NRLP3 inhibitor), bortezomib (NFKb inhibitor), Cx-01 (TLR-4 inhibitor), or OPN-305 (TLR-2 inhibitor) are actually being evaluated in early-stage clinical studies for MDS and AML [50,128,129,130,131,132]. Other targets, including S100A9, CD33, and IL-1 Receptor Accessory Protein will be studied.

### 7.4. Checkpoint Inhibitors

Following the successes with ICPi in solid tumors, these therapies are being evaluated in hematologic malignancies, including AML and MDS [133]. The first ICPi molecules challenged in clinical trials for HR-MDS patients are ipilimumab [134,135,136] (CTLA-4 inhibitor), nivolumab [137,138] or pembrolizumab [139,140] (PD-1 inhibitors), and atezolizumab or durvalumab [141,142,143] (PD-L1 inhibitors). Targeting TIGIT, CD70, or TIM-3 also seems to be an interesting option [84,85,144,145,146]. Response rates in monotherapy approaches are actually very low and combined strategies are more promising, especially with HMA. Indeed, treatment with HMA has been shown to upregulate several of these ICPs (such as PD-L1, TIM-3, and CD47) on MDS cells [77]. Moreover, the association of two ICPis (ipilimumab and nivolumab) has been also evaluated, finding the same efficacy as the results for monotherapy [135]. More recently, new ICPs have been identified as CD47, especially expressed by macrophages and acting as a “do not eat me” signal [147,148,149]. MDS cells from HR-MDS patients overexpress CD47 and this is associated with poor survival. Thus, the CD47–SIRPa interaction could be targeted by specific inhibitors [150]. Two anti-CD47 inhibitors have been evaluated in HR-MDS patients. First, CC-90002 was tested in phase 1, but was associated with a major toxicity profile and poor preliminary efficacy [151]. Another CD47 inhibitor, Magrolimab (HuF9-G4 or 5F9), seems to be less toxic than CC-90002 in a phase I trial [152]. Investigators reported high efficacy of magrolimab in association with HMA and a phase III evaluation of this association is currently in the recruitment stage (NCT04313881) [152].

Together, ICPis showed moderate and variable efficacy in early-phase trials with HR-MDS patients. The association with HMA seems to be more interesting than monotherapy, especially in the front line. Responders must be characterized more to identify the predictive factors of response with each ICPi.

### 7.5. Targeting NK-Cells

Similar to T-cells, NK-cells can also be exhausted in HR-MDS with the loss of their cytotoxic capacity [85,153,154]. Restoring their functional activity could help to avoid MDS progression. To address this hypothesis, new molecules are actually being evaluated, such as GTB-3550 TriKE™, a novel CD16/IL-15/CD33 Tri-Specific Killer Engager (TriKE) [155,156]. Pre-clinical data show specific NK cell activation and targeted cytolytic activity in xenogeneic AML mouse models. Another way to improve NK cell activity consists of blocking interactions between KIRs and human leukocyte antigen-C (HLA-C) molecules. Lirilumab is a human IgG4 monoclonal antibody that blocks KIR/HLA-C interaction [157].

### 7.6. Vaccination Strategies

Several tumor-associated antigens (TAA) have been identified and found to be overexpressed in MDS patients, leading to the development of specific vaccines to stimulate tumor-specific T cells [158,159,160]. TAAs targeted by peptide vaccines include Wilm’s tumor-1 (WT1), Proteinase-3+ neutrophil elastase (PR-1), or NY-ESO-1. The expression of WT1 is correlated with the marrow blast percentage, poor response to HMA, and poor OS [159,161,162,163,164]. The first studies of the peptide vaccine of WT1 have demonstrated an acceptable safety profile, good biological response (expansion of WT1 reactive T cells), but with no significant sustained clinical response [159,165,166,167,168]. PR-3 is another potential target, and a PR-1 vaccine (an HLA-A2-restricted peptide-based PR3 in combination with neutrophil elastase) was recently evaluated with the same profile of response as the WT1 vaccine [160]. NY-ESO1, a cancer testis antigen, has been found expressed on some solid tumors [169]. NY-ESO1 expression is low in MDS due to the silencing of TAA genes by the methylation mechanism [170]. As expected, early studies showed an increase in NY-ESO1 expression after treatment with HMA [171] and the results in MDS patients are encouraging [172]. New multiepitope vaccines are also being evaluated in combination with HMA, but the first results are disappointing (negative results and AML progression) [173].

To conclude, vaccine strategies in MDS have actually shown a good safety profile and interesting biological results but with poor clinical responses. Because dendritic cells play a key role in vaccine response and because these cells present some dysfunction in MDS patients [64,67,174], it could be important to consider this issue. Other vaccine strategies are also evaluated [175].

### 7.7. Adoptive T-Cell Transfer Therapy

Chimeric antigen receptor: MDS cells have been shown to overexpress the ligands for Natural killer group 2 (NKG2D) receptors [176,177], and clinical trials with NKG2D receptors CAR-T cells have been evaluated, but without favorable outcomes [178]. The expansion process could be improved by lymphodepleting chemotherapy prior to infusion, but a major issue seems to be the inability of CAR-T to persist as memory lymphocytes. Other studies evaluating new CAR-T cells are ongoing [179,180]. Because CD123 delineates MDS stem cells in HR-MDS patients, CD123 CARs have been generated and have demonstrated promising results in vitro and in vivo [181].

Donor-directed lymphocytes: While HSCT is the only curative treatment for MDS, relapses can occur and are associated with poor outcomes. In these cases, unmanipulated DLIs may be an option but these are associated with severe GVHD. DLI could be stimulated ex vivo by TAA, which selects for an enriched, polyclonal CD4+ and CD8+ specifically directed against MDS. TTA-DLI appears to be safer, with respect to GVHD, than unselected DLI [182,183].

**Table 1:** Current and potential immune targets in MDS.

	Molecule	Patients	NCT/Phase/Status	Ref.
Cytokines				
TGF-β	Luspatercept	LR-MDS with anemia	NCT02604433/Phase III/Authorization	[126]
TNF-α	Etanercept	HR-MDS (+Aza)	NCT00118287/I-II/Completed	[122]
IL-1	Canakinumab	LR-MDS (+Aza) LR-MDS (+luspa, +TIM3inh)	NCT04239157/II/Recruiting NCT04810611/Ib/Recruiting	
Inflammasome				
IRAK-4	CA-4948	HR-MDS (+aza and/or Ven)	NCT04278768/I-II/Recruiting	[128]
NLRP3	ibrutinib	HR-MDS (+Aza) HR-MDS (+Len)	NCT02553941/I/Recruiting NCT03359460/I/Recruiting	[129]
TLR				
TLR-4	CX-01	HR-MDS (+Aza)	NCT02995655/I/Completed	[131]
TLR-2	OPN-305	LR-MDS	NCT02363491/I-II/Completed	[132]
NF-kB	Bortezomib	HR-MDS (+Len)	NCT00580242/I/completed	[130]
MDSC				
CD33 inh	BI 836858	LR-MDS	NCT02240706/II/Terminated	
Immune check-points				
CTLA-4	ipilimumab	HR-MDS HR-MDS (and/or nivolumab +/−Aza) HR-MDS (+Dec)	NCT01757639/I/Completed NCT02530463/II/recruiting NCT02890329/I/Recruiting	[134,135]
PD-1	Nivolumab Prembrolizumab PDR001	HR-MDS (and/or ipi +/−Aza) HR-MDS (+chemo) HR-MDS (+Aza) HR-MDS(+Dec) HR-MDS (+Aza+/Tim3 inh)	NCT02530463/II/recruiting NCT02464657/II/completed NCT03094637/II/recruiting NCT03969446/I/recruiting NCT03066648/I/active	[135,137,139]
PD-L1	Durvalumab Atezolizumab	HR-MDS (+Aza) HR-MDS (+Guadecitabine) HR-MDS (+/−Aza)	NCT02775903/II/active NCT02935361/I-II/active NCT02508870/I/completed	[141,143,179]
TIM3	Sabatolimab (MBG453)	HR-MDS (+Aza+/−PD-1 inh) HR-MDS (+Aza) HR-MDS (+Aza+Ven)	NCT03066648/I/active NCT04266301/III/Recruiting NCT04812548/II/recruiting	[145,146]
CD70	Cusatuzumab (ARGX-110)	HR-MDS (+Aza) HR-MDS (+Aza)	NCT04241549/I/Active NCT03030612/I-II/Active	[146]
CD47	AK117 Hu5F9-G4 CC-90002 ALX148 Magrolimab	HR-MDS (+Aza) HR-MDS HR-MDS HR-MDS (+Aza) HR-MDS (+/−Aza) HR-MDS (+Aza)	NCT04900350/I-II/Recruiting NCT02678338/I/completed NCT02641002/I/Terminated NCT04417517/I-II/recruiting NCT03248479/I/completed NCT04313881/III/recruiting	[152,153]
NK-cells				
CD16/IL-15/CD33	GTB-3550 TriKE™	HR-MDS	NCT03214666/I-II/recruiting	[156]
KIR inh	Lirilumab	HR-MDS (+/−Aza)	NCT02599649/II/terminated	[157]
CAR-T-cells				
Cyad-O1/02	Cyad-O1/02	HR-MDS	NCT03466320/I-II/completed, NCT04167696/I/recruiting	[178]
NKX101	NKX101	HR-MDS	NCT04623944/I/Recruiting	[179]
Prgn-3006	Prgn-3006	HR-MDS	NCT03927261/I/recruiting	[180]
Vaccine				
	DSP-7888	LR and HR-MDS	NCT02436252/I-II/completed	[158]
	K562/GM-CSF/CD40L	HR-MD	NCT00840931/I/Completed	[175]
	NPMW	HR-MDS	NCT02750995/I/completed	[173]

Aza: Azacitidine; CD: Cluster of Differentiation; CTLA-4: Cytotoxic T-Lymphocyte Antigen 4; HR-MDS: High-risk myelodysplastic syndrome; IL: Interleukin; inh: Inhibitor; Len: Lenalidomide; luspa: Luspatercept; LR-MDS: Low-risk myelodysplastic syndrome; MDSC: Myeloid-Derived Suppressor Cells; NKG2D: Natural killer group 2 member; NLRP3: NOD-like receptor family, pyrin domain containing 3; PD-1: Program cell death-1; PD-L1: Program cell death-Ligand; TIM3: T-cell immunoglobulin and mucin containing protein-3; TGF-*β:* Transforming Growth Factor-*β*; TLR: Toll Like Receptor; TNF-α: Tumor Necrosis Factor-α; Ven: Venetoclax.

## 8. Conclusions

**Figure 1:**
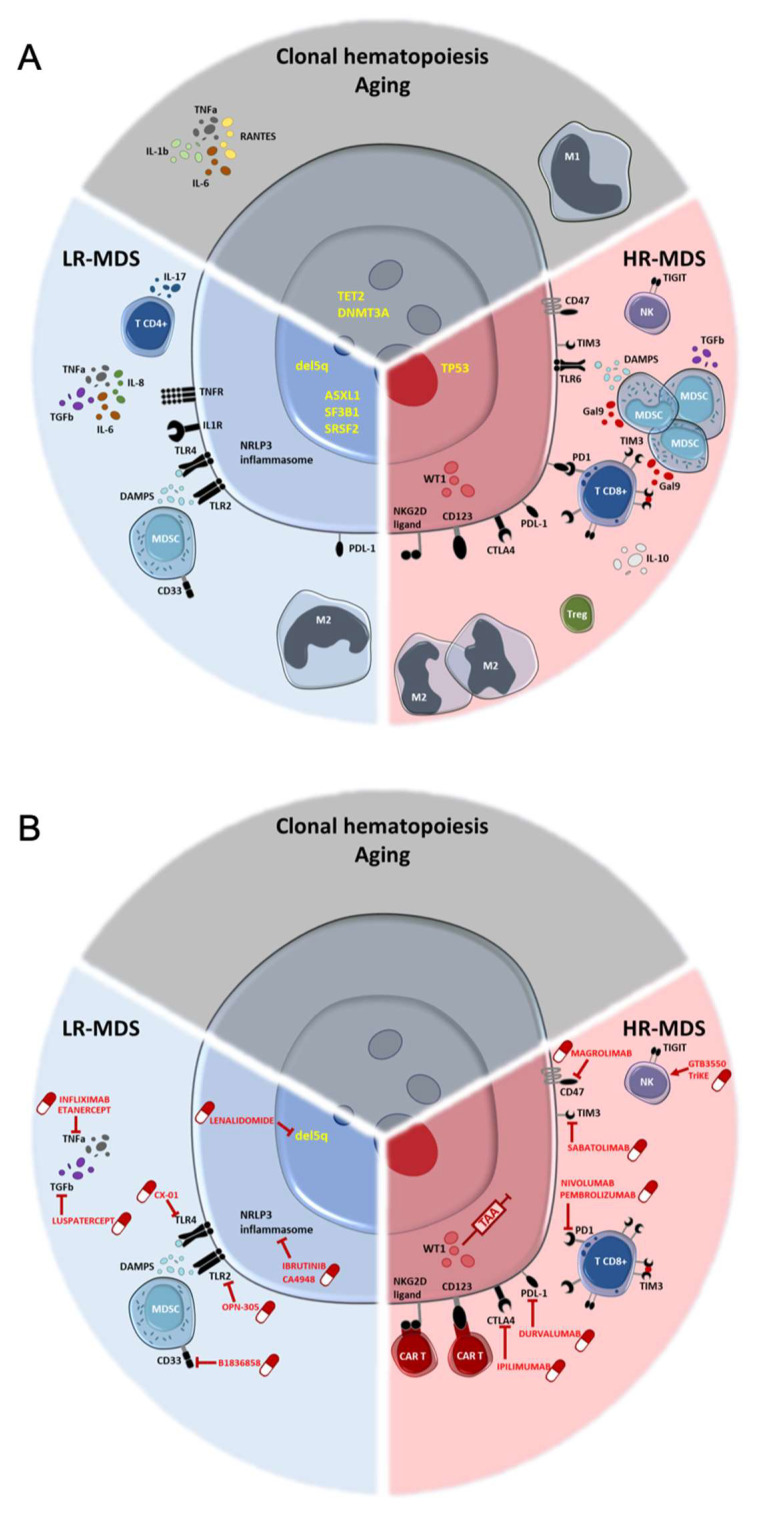
From immune dysregulations to therapeutic perspectives in myelodysplastic syndromes. (**A**) Immune key hubs involved in early stages, low-risk MDS, and high-risk MDS. (**B**) Available and potential drugs targeting immune system in MDS. CD: Cluster of Differentiation; CTLA-4: Cytotoxic T-Lymphocyte Antigen 4; DAMPs: Damage Associated Molecular Patterns; Del5q: Deletion 5q; DNMT3A: DNA methyl-transferase 3A; Gal9: Galectin 9; HR-MDS: High-risk myelodysplastic syndrome; IL: Interleukin; LR-MDS: Low-risk myelodysplastic syndrome; M1/M2: Macrophages type 1 and 2; MDSC: Myeloid-Derived Suppressor Cells; NKG2D: Natural killer group 2 member; NLRP3: NOD-like receptor family, pyrin domain containing 3; PD-1: Program cell death-1; PD-L1: Program cell death-Ligand 1; RANTES: Regulated upon Activation, Normal T Cell Expressed and Presumably Secreted; SF3B1: Splicing Factor 3b Subunit 1; SRSF2: Serine and Arginine Rich Splicing Factor 2; TET2: Ten-eleven-translocation 2; TIGIT: T cell immunoreceptor with Ig and ITIM domains; TIM3: T-cell immunoglobulin and mucin containing protein-3; TGF-*β*: Transforming Growth Factor-β; TLR: Toll Like Receptor; TNF-α: Tumor Necrosis Factor-α; TNFR: Tumor Necrosis Factor Receptor; TP53: Tumor Protein 53; Treg: Lymphocytes T regulators; WT1: Wilms Tumor 1.

Recent interest for alterations in the immune system in MDS has led to an improved understanding of its pathogenesis (Figure 1). Both the innate and adaptive immune systems have been shown to participate in MDS pathogenesis and progression, which has allowed the development of different therapeutic approaches in LR-MDS and HR-MDS. However, results are heterogeneous and future investigations should further explore the MDS immune landscape. In parallel, future studies will have to determine which patients will respond to a predefined treatment, in order to achieve a personalized approach.
